# Efficacy and Safety of Home-Based Cardiac Telemonitoring Rehabilitation in Patients After Transcatheter Aortic Valve Implantation: Single-Center Usability and Feasibility Study

**DOI:** 10.2196/45247

**Published:** 2023-05-17

**Authors:** Kohei Ashikaga, Shunichi Doi, Kihei Yoneyama, Norio Suzuki, Shingo Kuwata, Masashi Koga, Naoya Takeichi, Satoshi Watanabe, Masaki Izumo, Keisuke Kida, Yoshihiro J Akashi

**Affiliations:** 1 Department of Sports Medicine St. Marianna University School of Medicine Kawasaki Japan; 2 Department of Cardiology St. Marianna University School of Medicine Kawasaki Japan; 3 Rehabilitation Center St. Marianna University School of Medicine Hospital Kawasaki Japan; 4 Department of Pharmacology St. Marianna University School of Medicine Kawasaki Japan

**Keywords:** transcatheter aortic valve implantation, telerehabilitation, cardiac rehabilitation, remote, telemonitoring

## Abstract

**Background:**

No consensus exists on the efficacy of home-based cardiac rehabilitation (CR) in patients who have undergone transcatheter aortic valve implantation (TAVI). Additionally, there are no reports on home-based cardiac telemonitoring rehabilitation (HBTR) in patients after TAVI.

**Objective:**

We aimed to investigate the efficacy of HBTR in patients who have undergone TAVI.

**Methods:**

This single-center preliminary study introduced HBTR to patients after TAVI, and the efficacy outcomes of the rehabilitation method were compared to that of a historical control cohort. The historical control cohort (control group) consisted of 6 consecutive patients who underwent ordinary outpatient CR after TAVI from February 2016 to March 2020. Patients who participated in the HBTR program were only recruited after the TAVI procedure and before discharge between April 2021 and May 2022. In the first 2 weeks after TAVI, patients underwent outpatient CR and were trained using telemonitoring rehabilitation systems. Thereafter, patients underwent HBTR twice a week for 12 weeks. The control group performed standard outpatient CR at least once a week for 12 to 16 weeks. Efficacy was assessed using peak oxygen uptake (VO_2_) prior to and after CR.

**Results:**

Eleven patients were included in the HBTR group. All patients underwent 24 HBTR sessions during the 12-week training period, and no adverse events were observed. The control group participants performed 19 (SD 7) sessions during the training period, and no adverse events were observed. Participants in the HBTR and control groups had a mean age of 80.4 (SD 6.0) years and 79.0 (SD 3.9) years, respectively. In the HBTR group, preintervention and postintervention peak VO_2_ values were 12.0 (SD 1.7) mL/min/kg and 14.3 (SD 2.7) mL/min/kg (*P*=.03), respectively. The peak VO_2_ changes in the HBTR and control groups were 2.4 (SD 1.4) mL/min/kg and 1.3 (SD 5.0) mL/min/kg (*P*=.64), respectively.

**Conclusions:**

Home-based CR using a telemonitoring system is a safe outpatient rehabilitation method. Its efficacy is not inferior to that of standard CR in patients who have undergone TAVI.

**Trial Registration:**

Japan Registry of Clinical Trials jRCTs032200122; https://jrct.niph.go.jp/latest-detail/jRCTs032200122

## Introduction

Transcatheter aortic valve implantation (TAVI) was developed as a new catheter-based treatment for severe aortic valve stenosis. TAVI is a less invasive treatment; however, since majority of the patients who undergo TAVI are geriatric, reports have suggested that approximately half of all patients with intermediate risk who undergo TAVI are at risk of death or disability due to stroke within 5 years [1]. These adverse events after TAVI are associated with preoperative and perioperative physical dysfunction [2,3]. Postoperative cardiac rehabilitation (CR) is therefore crucial. Studies have indicated that post-TAVI CR improves exercise tolerance and reduces mortality [4,5]. However, the percentage of cardiac patients participating in outpatient CR is less than 10% in Japan, which poses a serious problem [6,7]. The low availability of practical and social support is likely related to the low participation rates [8,9]. Additionally, the lower number of patients undergoing TAVI owing to older age and a decline in their physical condition may have contributed to the low percentage of participation. Indeed, only 6 (2%) patients participated in outpatient CR at St. Marianna University Hospital among 390 patients who underwent TAVI.

In light of these considerations, home-based cardiac telemonitoring rehabilitation (HBTR) is considered a practical method for increasing participation in outpatient rehabilitation. HBTR is considered effective as a commute-less rehabilitation, and with this approach, the participation rate can possibly be increased. Some studies have reported HBTR in patients after TAVI [10-13]. One of these studies revealed the safety of HBTR in patients who have undergone TAVI [10]; however, no consensus has been reached regarding its efficacy, and all these studies were only performed using mobile apps or wearable devices. Therefore, the effectiveness of HBTR, and the safety and feasibility of HBTR in patients who have undergone TAVI remain unknown.

This study hypothesizes that HBTR is a feasible, safe, and effective approach to perform telemonitoring with appropriate support in patients who have undergone TAVI, and aims to investigate the efficacy of HBTR in patients who have undergone TAVI.

## Methods

### Study Design and Participants

This was a single-center preliminary study that introduced an HBTR program to patients after TAVI, with a historical control cohort. From April 2021 to May 2022, patients who underwent TAVI for aortic valve stenosis at St. Marianna University Hospital were recruited to participate in an HBTR program (HBTR group) in this study. Patients who participated in the HBTR program were only recruited after undergoing the TAVI procedure and before discharge according to the inclusion and exclusion criteria (Textbox 1).

The historical control cohort (control group) consisted of all 6 patients who underwent standard outpatient CR at the same institution out of 390 patients who underwent TAVI between February 2016 and March 2020.

**Inclusion and exclusion criteria.**

**Inclusion criteria**

1. Underwent transcatheter aortic valve implantation (TAVI) and started cardiac rehabilitation (CR) during hospitalization.
2. Provided written informed consent.
3. Aged over 20 years.
4. Can be accompanied by an attendant when remote CR is performed.

**Exclusion criteria**

1. New York Heart Association (NYHA) class 4.
2. Cerebral infarction after TAVI.
3. Myocardial infarction within 1 month of treatment.
4. Unknown or untreated syncope or cardiac arrest.
5. History of operation for implantable cardioverter defibrillator/cardiac resynchronization therapy-defibrillator, or cardiopulmonary arrest within the last 6 months.
6. Unstable angina pectoris.
7. Severe adverse events during hospitalized CR.
8. Severe renal dysfunction (estimate glomerular filtration rate <15 mL/min/1.73 m^2^).
9. Severe liver dysfunction.
10. Difficulty understanding the system of remote CR.
11. No internet connection at home.
12. Already participated in other clinical trials.
13. Cannot understand the contents of this trial due to dementia or other psychiatric diseases.
14. Participation deemed inappropriate by the research director.

### TAVI Procedure

Indications for TAVI were determined based on current recommendations [14]. An interdisciplinary heart team, including cardiothoracic surgeons, anesthesiologists, interventional cardiologists, and echocardiography cardiologists, selected the valve type and decided upon other procedural strategies. TAVI was performed in a hybrid operating room under general anesthesia. As part of general care, all patients underwent standardized inpatient CR after TAVI.

### Ethics Approval

Written informed consent for publication of their details was obtained from the study participants. This study was performed in accordance with the ethical principles of the Declaration of Helsinki. This study was also approved by the Clinical Research Ethics Committee of St. Marianna University School of Medicine (study protocol number: SMU0124), and the study was registered with the Japan Registry of Clinical Trials (jRCTs032200122) on September 14, 2020. Additionally, an independent data safety monitoring board reviewed the patient data.

### Cardiopulmonary Exercise Test

**Figure 1:**
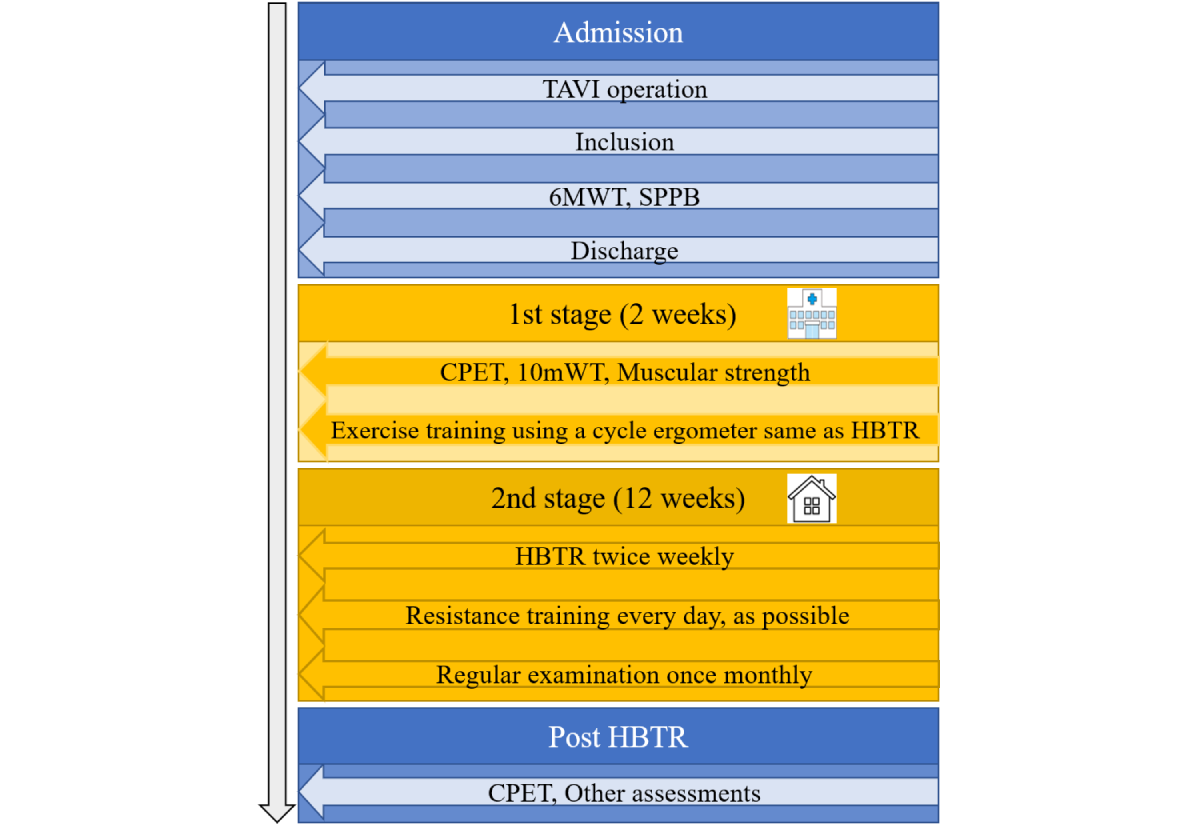
Timeline of the intervention in the HBTR group. Patients were recruited and included after the TAVI procedure. In the first stage, for 2 weeks, the CPET, 10mWT, and muscular strength were simultaneously assessed. After these assessments, the participants practiced with the cycle ergometer and telemonitoring system, which were the same as those in HBTR, in the hospital. In the second 12-week stage, the participants performed HBTR twice weekly. At the end of the second 12-week stage, the CPET, 6MWT, SPPB, 10mWT, and muscular strength were assessed within 2 weeks after the last HBTR session. 6MWT: 6-minute walk test; 10mWT: 10-m walk test; CPET: cardiopulmonary exercise test; HBTR: home-based cardiac telemonitoring rehabilitation; SPPB: short physical performance battery; TAVI: transcatheter aortic valve implantation.

For the preintervention and postintervention physical assessments, symptom-limited cardiopulmonary exercise tests (CPETs) were performed to determine peak oxygen uptake (VO_2_), anaerobic threshold (AT), and carbon dioxide production efficiency derived from the linear relationship between minute ventilation (VE) and carbon dioxide output (VCO_2_) (VE vs VCO_2_ slope) using a cycle ergometer (SE-8; Mitsubishi Electric Engineering Co, Ltd) and a breath-by-breath gas analyzer (Inter Reha Co, Ltd). The exercise protocol for the cycle ergometer involved a 0-W warm-up and 10-W/min ramping. The preinterventional CPET was performed when starting the first stage of the rehabilitation program, and the postinterventional CPET was performed within 2 weeks after the final session of HBTR (Figure 1). In the control group, the preinterventional CPET was performed within 2 weeks after discharge and the postinterventional CPET was performed within 2 weeks after the final session of outpatient rehabilitation.

### Physical Assessment

The 6-minute walk test (6MWT) and short physical performance battery (SPPB), which is composed of a composite of 4-meter walking velocity, time taken to rise from a seated position 5 times, and standing balance, were performed immediately before discharge and within 2 weeks after the last session of HBTR. The 10-meter walk test (10mWT) and muscular strength were examined on the same day as the CPET. The 10mWT was performed in 2 different ways. Initially, the participant walked at a comfortable speed, and the second time, the participant walked as quickly as possible. This test assessed the participant’s gait speed (m/s). Muscular strength was assessed by measuring hand grip strength (HGS) and quadriceps isometric strength (QIS). The HGS was measured using a grip meter (JAMAR; Bissell Healthcare Co). The QIS was measured using a digital handheld dynamometer (µ-Tas; ANIMA). The HGS and QIS values were defined as the average values of the left and right limbs. In the control group, the SPPB and 10mWT were performed just before discharge from the hospital and within 2 weeks after the final session of outpatient rehabilitation. Muscular strength was measured on the same day as the CPET. However, the 6MWT was not performed in the control group.

### Intervention

The intervention participants underwent a 14-week hybrid CR program consisting of 2 stages in the HBTR group. In the 2 weeks of the first stage, a baseline clinical examination was performed, and patients were educated as part of a comprehensive program. The participants also practiced on a cycle ergometer (ai-ex; Konami Sports & Life Co, Ltd). Simultaneously, they were familiarized with the telemonitoring system (Heart-Line; Nipro Co, Ltd), which was the same as that used in HBTR. The participants were trained to become accustomed to these technologies at least twice during the first stage. After the first stage, a cycle ergometer and a tablet PC (iPad; Apple Co, Ltd) were delivered by a mechanical supervisor within 2 weeks. During this period, participants performed standard outpatient rehabilitation 1 to 2 times a week.

In the second 12-week stage, participants in the HBTR group performed HBTR twice weekly. These participants performed aerobic training using the cycle ergometer. The target intensity was based on the AT from the CPET at the start of the second stage. Before starting the exercise, medical staff had video calls with participants. The medical staff assessed the participants’ physical state, and the participants began the exercise thereafter. The video call was maintained throughout the exercise session, and the medical staff checked the electrocardiogram (ECG) via the internet. The exercise duration was initially at 15 minutes and was gradually increased to 30 minutes within the first 2 weeks. The exercise load was arranged according to the participant’s perceived exertion (ie, a score of 11-13 on the Borg scale).

Additionally, participants were instructed to perform 3 sets of 10 repetitions of resistance training (standing calf raises and sit-to-stand exercises) every day. The medical staff checked whether the participants were able to perform the resistance training every day during every video call.

In contrast, the control group performed standard outpatient CR once to twice a week for 12 to 16 weeks after TAVI. CR consisted of aerobic exercise using a cycle ergometer and treadmill ergometer, and mild-to-moderate resistance training. The intensity of aerobic exercise was based on the AT from the CPET at the start of outpatient CR. The exercise time was half an hour to 1 hour per session. In addition to the usual outpatient CR, the control group participants were also instructed to perform 3 sets of 10 repetitions of resistance training (standing calf raises and sit-to-stand exercises) every day.

During the rehabilitation term, patients in both groups were examined in the hospital once a month.

### Telemonitoring Rehabilitation Equipment and Management

**Figure 2:**
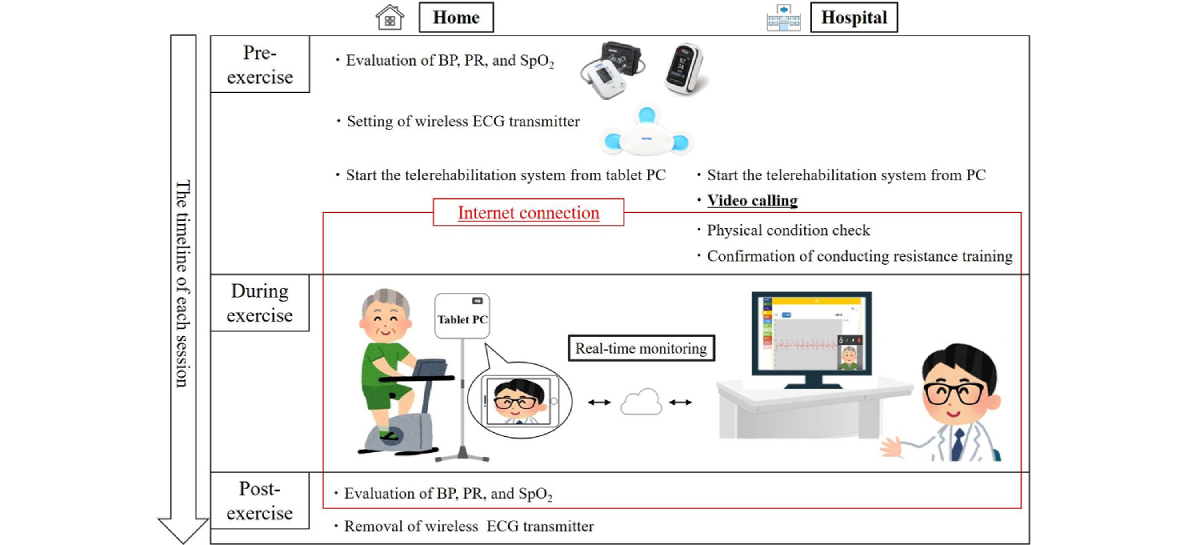
HBTR session timeline. Before each exercise session, the participants’ BP, PR, and SpO_2_ were evaluated. Soon after, a wireless ECG transmitter was placed on the left precordial side of the chest, and a telerehabilitation app on a tablet PC was initiated. Thereafter, all participants waited for video calls from the medical staff. The medical staff started video calling after launching the telerehabilitation app. During the video call, the medical staff evaluated the participants’ physical conditions and confirmed the implementation status of resistance training. Subsequently, the participants began the exercise. During the exercise, the medical staff continued to check the participants and monitor their ECG data. After the exercise, the participants re-evaluated their BP, PR, and SpO_2_. The video call was ended after the medical staff confirmed these parameters. The participants then removed their wireless ECG transmitters. BP: blood pressure; ECG: electrocardiogram; PR: pulse rate; SpO_2_: percutaneous oxygen saturation.

For the exercise training, all participants used the same type of cycle ergometer. Before and after each exercise session, each participant measured their blood pressure, pulse rate, and percutaneous oxygen saturation (SpO_2_) using a blood pressure manometer (NBP-1BLE; Nipro Co, Ltd) and a pulse oximeter (MightySat; Nipro Co, Ltd) (Figure 2). Before starting each aerobic exercise session, the participants put on a wireless ECG transmitter (Cocolon; Nipro Co, Ltd) and opened the telemonitoring app from the tablet PC. To simplify this task, the tablet setup only allowed the patients to use the telemonitoring app. At the start of the aerobic exercise session, video calling was performed by rehabilitation medical staff at the hospital using a telemonitoring app system. During exercise training, video and ECG monitoring were continued using the telemonitoring app system. This monitoring system was encrypted using a secure socket layer.

In this study, all participants were required to be accompanied by an attendant at each exercise session in anticipation of adverse events. During the first 2 to 4 exercise sessions, the mechanical supervisor assisted with the operation of each piece of equipment. At the time of each exercise session, if the participant could not connect to the telemonitoring app system, the session was moved to another day, and the medical staff or mechanical supervisor assisted with the connection until the next session.

### Other Measurements

We examined patient baseline characteristics and the Society of Thoracic Surgeons (STS) risk scores for predicting the risk of mortality [15,16]; used the Mini Nutritional Assessment-Short Form (MNA-SF) for assessing preoperative nutritional status [17]; and assessed procedural outcomes, duration of postoperative hospitalization, laboratory data, and medication. Laboratory data were assessed just before discharge from the hospital and on the same day as the postinterventional assessment. Medication was assessed at the time of discharge. The Katz index was used to assess and record basic activities of daily living and functional status, which were evaluated at discharge from the hospital [18]. Information concerning the success of the implanted device was obtained from the Valve Academic Research Consortium-2 criteria [19]. Early safety was evaluated 30 days post-TAVI by assessing the procedural outcomes of all-cause death, stroke (disabling and nondisabling), life-threatening bleeding, acute kidney injury (risk, injury, failure, loss, and end-stage kidney disease [stage 2 or 3, or renal replacement therapy]), coronary artery obstruction requiring intervention, major vascular complications, and pacemaker implantation after TAVI. The duration of postoperative hospitalization was defined as the time from operation to discharge. Laboratory data were evaluated at the first session of the first stage, and medication was evaluated at discharge.

### Endpoint

The primary endpoint was the change in peak VO_2_ between the initial and final CPET. The secondary endpoints were the changes in AT, 6MWT, grip strength, and isometric knee extension force. Additionally, during the 12-week rehabilitation, the safety of HBTR was evaluated by assessing adverse events during exercise training.

### Statistical Analysis

Baseline characteristics, physical assessments, and CPET data between preintervention and postintervention were compared in the HBTR group. Additionally, changes in preintervention and postintervention values were compared between the HBTR and control groups.

Continuous variables have been expressed as mean (SD), and categorical variables have been expressed as numbers and percentages. The normality of distribution for continuous variables was evaluated using the Shapiro-Wilk test. The Mann-Whitney *U* test was used to analyze quantitative variables, and the Fisher exact test was used for qualitative variables. Statistical significance was set at a 2-sided *P-*value <.05. All analyses were performed using JMP Pro version 15 (SAS Institute).

## Results

### Patient Characteristics

**Figure 3:**
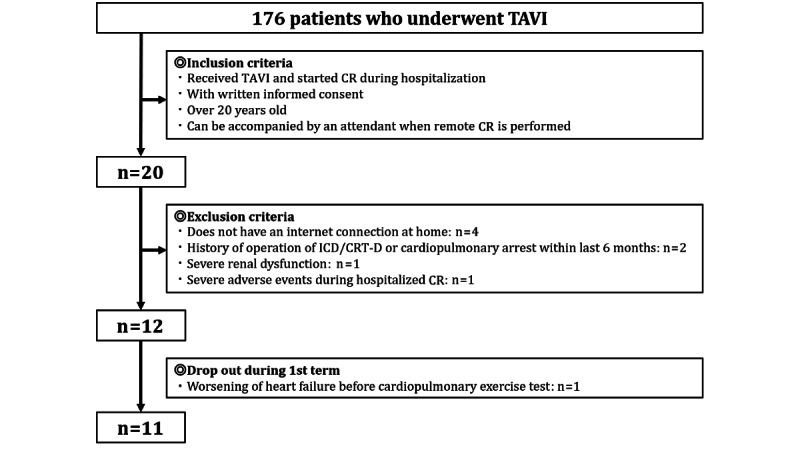
Study flowchart of the HBTR group. CR: cardiac rehabilitation; CRT-D: cardiac resynchronization therapy-defibrillator; HBTR: home-based cardiac telemonitoring rehabilitation; ICD: implantable cardioverter defibrillator; TAVI: transcatheter aortic valve implantation.

In the HBTR group, 176 patients underwent TAVI. Of these, 164 patients met the exclusion criteria or failed to meet the inclusion criteria. Of the 12 patients who met the inclusion criteria, 11 patients completed the first 2-week stage; 1 patient did not undergo HBTR due to worsening heart failure before starting stage 1 CR (Figure 3).

In the control group, all 6 patients met all the inclusion criteria except criterion number 4 and they did not meet criteria numbers 1 to 10 and 12 to 14 of the exclusion criteria (Textbox 1). The control group participants performed 19 (SD 7) sessions during the training period.

With regard to the second 12-week stage, there were 8 occasions where the exercise session was prolonged because of internet connection errors; however, all 11 patients underwent 24 HBTR sessions. Patient demographics and clinical characteristics at baseline are shown in Table 1. The mean patient age was 80.4 (SD 6.0) years and 79.0 (SD 3.9) years in the HBTR and control groups, respectively. Three participants out of 11 in the HBTR group and 3 out of 6 in the control group had an MNA-SF score of less than 12 points. There were no significant differences in patient characteristics between the HBTR and control groups.

**Table 1:** Baseline patient characteristics.

Characteristic		HBTR^a^ group (n=11)	Control group (n=6)	*P* value^b^
Age (years), mean (SD)		80.4 (6.0)	79.0 (3.9)	.34
Male gender, n (%)		6 (55)	3 (50)	.87
BMI (kg/m^2^), mean (SD)		26.1 (4.6)	23.7 (3.1)	.21
**NYHA^c^, n (%)**				.49
	I	9 (82)	4 (67)	
	II	2 (18)	2 (33)	
	III	0 (0)	0 (0)	
Duration of postoperative hospitalization, mean (SD)		6.2 (1.7)	7.7 (2.1)	.17
Hypertension, n (%)		10 (91)	5 (6)	.65
Dyslipidemia, n (%)		9 (82)	6 (100)	.09
Diabetes mellitus, n (%)		6 (55)	2 (33)	.40
COPD^d^, n (%)		2 (19)	2 (33)	.49
Previous pacemaker implantation, n (%)		1 (9)	1 (17)	.65
Atrial fibrillation, n (%)		5 (45)	1 (17)	.22
Previous cerebral infarction, n (%)		2 (19)	0 (0)	.17
Previous PCI^e^ or CABG^f^, n (%)		1 (9)	1 (17)	.22
Peripheral artery disease, n (%)		1 (9)	2 (33)	.07
Prior open cardiac surgery, n (%)		1 (9)	1 (17)	.65
Preoperative STS^g^ score (mortality), mean (SD)		3.6 (1.7)	4.1 (1.9)	.56
Katz index, mean (SD)		6.0 (0.0)	6.0 (0.0)	>.99
MNA-SF^h^, mean (SD)		11.5 (0.8)	12.7 (1.2)	.32
Hemoglobin level (g/dL), mean (SD)		11.2 (1.3)	12.4 (1.2)	.35
Albumin level (g/dL), mean (SD)		4.0 (0.3)	4.1 (0.2)	.69
eGFR^i^ (mL/min/1.73 m^2^), mean (SD)		53.2 (19.3)	51.4 (10.5)	.81
NT-proBNP^j^ (pg/mL), mean (SD)		852.5 (710.2)	500.0 (282.3)	.17
LVEF^k^ (%), mean (SD)		60.9 (4.2)	66.3 (5.1)	.05
Aortic valve area (cm^2^), mean (SD)		1.63 (0.26)	1.51 (0.47)	.57
Mean pressure gradient (mmHg), mean (SD)		10.1 (3.1)	13.2 (2.6)	.08
Transfemoral approach, n (%)		11 (100)	6 (100)	>.99
Device success, n (%)		10 (91)	6 (100)	.34
Early safety at 30 days, n (%)		10 (91)	6 (100)	.34
**Medication, n (%)**				
	β-blocker	4 (36)	4 (67)	.56
	ACE-I^l^/ARB^m^	8 (73)	4 (67)	.82
	Calcium channel blocker	5 (45)	4 (67)	.44
	Loop diuretics	4 (36)	1 (17)	.40
	Aspirin/clopidogrel	5 (45)	5 (83)	.12
	Warfarin	1 (9)	0 (0)	.34
	Direct oral anticoagulants	5 (45)	1 (17)	.23
	Statins	9 (82)	6 (100)	.17

^a^HBTR: home-based cardiac telemonitoring rehabilitation.

^b^The Mann-Whitney *U* test was used to analyze quantitative variables, and the Fisher exact test was used for qualitative variables.

^c^NYHA: New York Heart Association.

^d^COPD: chronic obstructive pulmonary disease.

^e^PCI: percutaneous coronary intervention.

^f^CABG: coronary artery bypass grafting.

^g^STS: Society of Thoracic Surgeons.

^h^MNA-SF: Mini Nutritional Assessment-Short Form.

^i^eGFR: estimated glomerular filtration rate.

^j^NT-proBNP: N-terminal pro-B natriuretic peptide.

^k^LVEF: left ventricular ejection fraction.

^l^ACE-I: angiotensin-converting enzyme inhibitor.

^m^ARB: angiotensin-receptor blocker.

### Efficacy of Cardiac Telerehabilitation

The preintervention and postintervention physical assessment outcomes in the HBTR group showed that the postinterventional peak VO_2_ and 6MWT values were significantly greater than the preinterventional values (mean 14.3, SD 2.7 mL/min/kg vs mean 12.0, SD 1.7 mL/min/kg; *P*=.03; and mean 345.0, SD 109.7 m vs mean 267.0, SD 72.0 m; *P*=.04, respectively; Table 2). Regarding other parameters, there were no significant changes between the preintervention and postintervention assessments.

**Table 2:** Changes in physical assessment outcomes in the study groups.

Characteristic			HBTR^a^ group, mean (SD)							Control group, mean (SD)							*P* value^b^ for peri-interventional difference in the HBTR group			*P* value^b^ for change between the 2 groups		
			Baseline		After intervention		Change		Baseline			After intervention		Change								
Peak VO_2_^c^ (mL/min/kg)			12.0 (1.7)		14.3 (2.7)		2.4 (1.4)		13.2 (1.6)			14.5 (5.2)		1.3 (5.0)		.03			.64			
6-minute walk test (m)			267.0 (72.0)		345.0 (109.7)		78.0 (83.5)		N/A^d^			N/A		N/A		.04			N/A			
AT^e^ (mL/min/kg)			8.7 (1.3)		9.6 (1.4)		0.9 (1.1)		9.2 (1.4)			9.9 (1.4)		0.6 (1.9)		.15			.61			
VE^f^ vs VCO_2_^g^ slope			33.0 (6.4)		30.4 (4.2)		−2.6 (4.5)		32.5 (6.9)			28.8 (8.2)		−3.7 (4.0)		.48			.63			
Peak work rate (watt)			54.3 (11.7)		62.7 (13.5)		8.5 (7.1)		60.0 (12.2)			64.2 (10.6)		4.2 (13.1)		.87			.48			
Peak RER^h^			1.17 (0.13)		1.16 (0.11)		0.01 (0.14)		1.11 (0.11)			1.17 (0.07)		0.06 (0.15)		.33			.35			
Hand grip strength (kg)			20.0 (7.5)		20.7 (7.1)		0.7 (2.2)		23.9 (3.2)			25.7 (5.3)		1.8 (2.6)		.82			.34			
Quadriceps isometric strength (kg)			24.7 (7.8)		26.0 (7.0)		1.3 (3.5)		23.6 (5.0)			25.7 (7.2)		2.1 (3.0)		.69			.35			
**SPPB^i^ (points)**			10.4 (12.2)		11.1 (1.6)		0.7 (1.4)		11.6 (0.5)			11.7 (0.5)		0.2 (0.4)		.31			.25			
	Balance	3.8 (0.4)		3.9 (0.3)		0.1 (0.3)		4.0 (0.0)			4.0 (0.0)		0.0 (0.0)		.56			.34				
	Gait speed	3.3 (0.8)		3.7 (0.6)		0.5 (0.8)		4.0 (0.0)			4.0 (0.0)		0.0 (0.0)		.15			.10				
	Chair stand	3.3 (1.1)		3.5 (1.2)		0.2 (0.8)		3.5 (0.5)			3.7 (0.5)		0.2 (0.4)		.72			.96				
**10-meter walk speed (m/s)**																						
	Comfortable	0.9 (0.2)		1.0 (0.2)		0.1 (0.2)		1.1 (0.1)			1.1 (0.1)		0.0 (0.2)		.32			.32				
	Fastest	1.2 (0.3)		1.3 (0.3)		0.1 (0.2)		1.4 (0.2)			1.4 (0.1)		0.0 (0.1)		.51			.43				

^a^HBTR: home-based cardiac telemonitoring rehabilitation.

^b^The Mann-Whitney *U* test was used to analyze quantitative variables.

^c^VO_2_: oxygen uptake.

^d^N/A: not applicable.

^e^AT: anaerobic threshold.

^f^VE: minute ventilation.

^g^VCO_2_: carbon dioxide output.

^h^RER: respiratory exchange ratio.

^i^SPPB: short physical performance battery.

### Change Values in the HBTR and Control Groups

The comparison of change values between the HBTR and control groups is shown in Table 2. The peak VO_2_ change values were 2.4 (SD 1.4) mL/min/kg and 1.3 (SD 5.0) mL/min/kg in the HBTR and control groups, respectively (*P*=.64). There were no significant differences in any of the variables between the 2 groups.

### Safety

No adverse events were observed in the HBTR and control groups.

## Discussion

### Principal Findings

This study investigated the efficacy and safety of an HBTR program involving the use of a cycle ergometer in patients after TAVI, with a historical cohort. In this study, all patients in the HBTR group completed all exercise sessions twice a week, and no adverse events were reported. HBTR was significantly effective in improving exercise tolerance after TAVI. Additionally, the efficacy of HBTR was comparable to that of standard outpatient CR.

### Effectiveness of CR for Patients After TAVI

In this study, our analysis showed similar changes in exercise tolerance, assessed by peak VO_2_, between the HBTR and control groups. In the HBTR group, although the peak VO_2_ and 6MWT values significantly improved, no significant difference in the change in muscle strength was observed. A previous study suggested that cardiac telerehabilitation improves lower muscle strength [20]. In contrast, our study participants were older than those in the previous study, and the previous study did not include patients who underwent TAVI, but instead included those with heart failure. Furthermore, the exercise frequency in our study was lower than that reported in the previous study [20]. These factors may have affected our results as aging is one of the main factors affecting skeletal muscle loss [21].

Another previous study that included patients who underwent TAVI showed that standard CR improves exercise tolerance, as assessed by the 6MWT [22]. Compared to the aforementioned study, the exercise frequency in this study was lower and our program duration was longer, yet we observed a similar effect (12 weeks of exercise did improve exercise tolerance).

### Telemonitoring Rehabilitation for Patients After TAVI

The low ratio of outpatient CR participants in Japan is related to low practical and social support [8,9]. One reason for this is associated with physical function; a decline in the physical function of a patient requires more support to visit a hospital. Most of the patients who underwent TAVI were geriatric, and our previous study showed that about two-thirds of patients who underwent TAVI were categorized as having physical prefrailty or frailty [23]. Therefore, patients who undergo TAVI are more likely to encounter difficulties in visiting a hospital unassisted. Thus, HBTR may be a solution for these types of patients.

Adapting HBTR for general use and preventing internet connection errors are the most important areas of this mode of rehabilitation. Internet connection errors usually occur due to slow connection speed or operation errors. With regard to connection, connecting to the telerehabilitation system entailed the use of video calling and also ECG monitoring. Therefore, to reduce the internet load, we did not conduct digital monitoring of blood pressure, pulse rate, and SpO_2_. With respect to operation errors, the average age of the participants in this study was 80 years, and low information technology literacy was assumed. Therefore, to reduce the risk of failing to complete the program, the presence of an attendant was required. We were able to finally complete full sessions of HBTR because of the attendants assisting the participants.

### Clinical and Research Scope of the Study in the Future

The HBTR program in this study mainly involved exercise therapy and patient education. We did not provide nutritional or dietary support. To achieve comprehensive CR, nutritional or dietary support is important in addition to exercise therapy and patient education. Low food intake is one of the main reasons for frailty, and steeper declines in food intake have been reported among even older adults [24]. Because of this, malnutrition is a prognostic factor in patients undergoing TAVI [23]. Therefore, we should have provided more nutritional or dietary support to the participants.

Future studies should include programs of exercise therapy combined with nutritional or dietary support for patients who are undergoing TAVI in larger and more diverse cohorts.

### Limitations

This study has several limitations. First, this was a single-center nonrandomized study with a small number of patients; thus, the possibility of type 1 error in the results of this study cannot be denied, and the generalizability of our findings is limited. Second, there may have been selection bias as the inclusion criteria of this study were limited by the needs of the participants to be supported by an attendant and to have an internet network; thus, patients with social and environmental vulnerabilities may have been excluded. Third, there may have been some information bias. In this study, daily activity could not be evaluated; we could therefore not exclude the possibility that daily activity affected the results. In addition, in the HBTR sessions, we could not monitor the cycle ergometer. Therefore, we could not confirm whether the patients had adjusted the device to the correct intensity.

### Conclusions

Our study results demonstrate that HBTR is effective in improving exercise tolerance and can be safely performed in patients who have undergone TAVI. However, this study had a small sample size; therefore, a further investigation is required to establish an optimal assessment of HBTR in this group of patients.

## Abbreviations

6MWT: 6-minute walk test
10mWT: 10-meter walk test
AT: anaerobic threshold
CPET: cardiopulmonary exercise test
CR: cardiac rehabilitation
ECG: electrocardiogram
HBTR: home-based cardiac telemonitoring rehabilitation
HGS: hand grip strength
MNA-SF: Mini Nutritional Assessment-Short Form
QIS: quadriceps isometric strength
SpO_2_: percutaneous oxygen saturation
SPPB: short physical performance battery
TAVI: transcatheter aortic valve implantation
VCO2: carbon dioxide output
VE: minute ventilation
VO2: oxygen uptake

## Data Availability

The deidentified participant data will not be shared.
